# Tendon versus Pyrocarbon Interpositional Arthroplasty in the Treatment of Trapeziometacarpal Osteoarthritis

**DOI:** 10.1155/2019/7961507

**Published:** 2019-07-22

**Authors:** Won-Taek Oh, Yong-Min Chun, Il-Hyun Koh, Jong-Kwan Shin, Yun-Rak Choi, Ho-Jung Kang

**Affiliations:** Department of Orthopedic Surgery, Yonsei University College of Medicine, 03722 Seoul, Republic of Korea

## Abstract

**Background:**

Trapeziometacarpal (TMC) arthritis is treated with surgery when nonsurgical treatment fails. The best surgical option for improving pain relief, functional outcomes, and postoperative complications remains controversial. The purpose of this study was to compare clinical and radiological outcomes and complications between trapezium excision with ligament reconstruction and tendon interposition (LRTI) and pyrolytic carbon interpositional arthroplasty.

**Methods:**

From March 2009 to August 2014, 37 patients (39 wrists) with Eaton-Littler stage II or III TMC arthritis underwent complete trapezium excision with LRTI (Group L, n=19) or pyrolytic interpositional arthroplasty (Group P, n=20). Visual analog scale (VAS) pain scores; grip and pinch strength; Kapandji scores to quantify thumb opposition; and Disabilities of Arm, Shoulder, and Hand (DASH) scores were used to compare clinical outcomes between the two groups. Radiographic changes (metacarpal shortening, subluxation, and radiolucency) were evaluated on the radiographs of thumb basal joints.

**Results:**

There were no differences in patient demographics, Eaton-Littler stage, preoperative outcome measures, or the duration of follow-up between the two groups. At the last follow-up, VAS pain scores, pinch and grip strengths, Kapandji scores, and DASH scores were significantly improved in both groups compared with preoperative scores. All follow-up measurements were similar between the two groups except pinch strength, which was 1.8 kg higher in Group P (p<0.001). Proximal metacarpal migration did not differ significantly between the groups. Periprosthetic lucency more than 1 mm was observed in 7 of 20 (35%) thumbs. Complication rates were similar between the two groups.

**Conclusions:**

All subjective and objective outcomes were similar following LRTI and pyrolytic interpositional arthroplasty in patients with TMC arthritis, except pinch strength, which was more improved following pyrolytic interpositional arthroplasty. Longer follow-up is required to test adverse effects of high rates of periprosthetic lucency and prosthetic subluxation on clinical outcomes after PyroDisk interpositional arthroplasty.

## 1. Introduction

The trapeziometacarpal (TMC) joint is a saddle joint with high mobility in three planes and is prone to the development of symptomatic osteoarthritis [1]. As the arthritis progresses, patients report constant pain with weakness, loss of movement, and deterioration of hand function [2]. Various surgical options are indicated based on the stage of progression to provide the patient with pain relief and functional improvement when conservative management fails [3]. Partial or complete trapezium resection with or without interposition or prosthesis implantation is recommended to preserve joint movement when radiographs show space narrowing and osteophytes.

Trapezium excision with ligament reconstruction and tendon interposition (LRTI), as described by Burton and Pellegrini [4, 5], is the traditional surgical option for TMC arthritis. Any basal joint technique that includes complete trapezium excision, however, may result in thumb shortening and reduced pinch strength. For example, Tomaino et al. [5] and Wang and Weiland [6] report limited improvement of pinch strength of 8% and 17%, respectively, following LRTI. Mechanical studies have shown that prosthesis implantation reduces metacarpal subsidence and better replicates normal joint motion [7–9]. Controversy exists regarding the surgical method for TMC arthritis that best improves pain and functional outcomes.

The pyrolytic carbon nonanatomical interposition implant (PyroDisk, Integra Life Sciences, Plainsboro, NJ, USA) has been used to restore biomechanics after partial resection of the joint in patients with advanced thumb basal joint arthritis [10–12]. Barrera-Ochoa et al. reported improved pain and weakness and a prosthesis survival rate of 90% at a minimum follow-up of 5 years [10]. Data on clinical and radiological outcomes and the types of complications following PyroDisk interpositional arthroplasty compared to trapezium excision with LRTI are sparse, however. The main aims of this study, therefore, were to compare clinical and radiological outcomes of PyroDisk interpositional arthroplasty to trapezium excision with LRTI in the surgical treatment of advanced TMC arthritis and to compare surgery-related complications between these two methods. We asked the following questions: (1) Is there a difference in clinical and radiological outcomes after trapezium excision with LRTI compared to pyrolytic carbon interpositional arthroplasty at a minimum follow-up of 2 years? (2) Is there a difference in perioperative or postoperative complications between these two surgical treatment options?

## 2. Materials and Methods

We retrospectively reviewed the records of all patients with Eaton-Littler stage II or III TMC arthritis who underwent trapezium excision with LRTI or PyroDisk interpositional arthroplasty [13] from March 2009 to August 2014 and had 2 or more years of follow-up. Surgical treatment was indicated following failure of nonsurgical treatments, including rest, splinting, nonsteroidal anti-inflammatory drugs, corticosteroid injections, and physiotherapy. Patients included in this study had no evidence of scaphotrapeziotrapezoidal arthritis, which was verified during surgery [13]. Patients meeting the following criteria were excluded: (1) Eaton-Littler stage I or IV TMC arthritis [13]; (2) traumatic TMC joint arthritis following fracture or dislocation around the joint; (3) TMC joint surgeries other than LRTI or PyroDisk arthroplasty; (4) any previous surgery on the involved upper limb; (5) worker's compensation issues; and (6) inadequate follow-up (<24 months). Patients who underwent trapezium excision with LRTI were classified as Group L and those who underwent PyroDisk interpositional arthroplasty were classified as Group P. One senior hand surgeon (YRC) performed two kinds of surgery alternatively under same surgical indications. Fifty-five patients met the initial inclusion criteria during the study period. Five patients with Eaton-Littler stage I or IV arthritis, two patients with TMC arthritis following comminuted metacarpal or trapezium fracture, four patients who underwent TMC arthrodesis, three patients with previous surgery on the involved limb, one patient receiving worker's compensation, and one patient who had inadequate follow-up were subsequently excluded. In total, 16 patients were excluded and 39 patients were enrolled in the study, 19 of whom underwent trapezium excision with LRTI and 20 of whom underwent PyroDisk arthroplasty. Our Institutional Review Board approved the study and waived the requirement for informed consent.

### 2.1. Surgical Techniques

**Figure 1:**
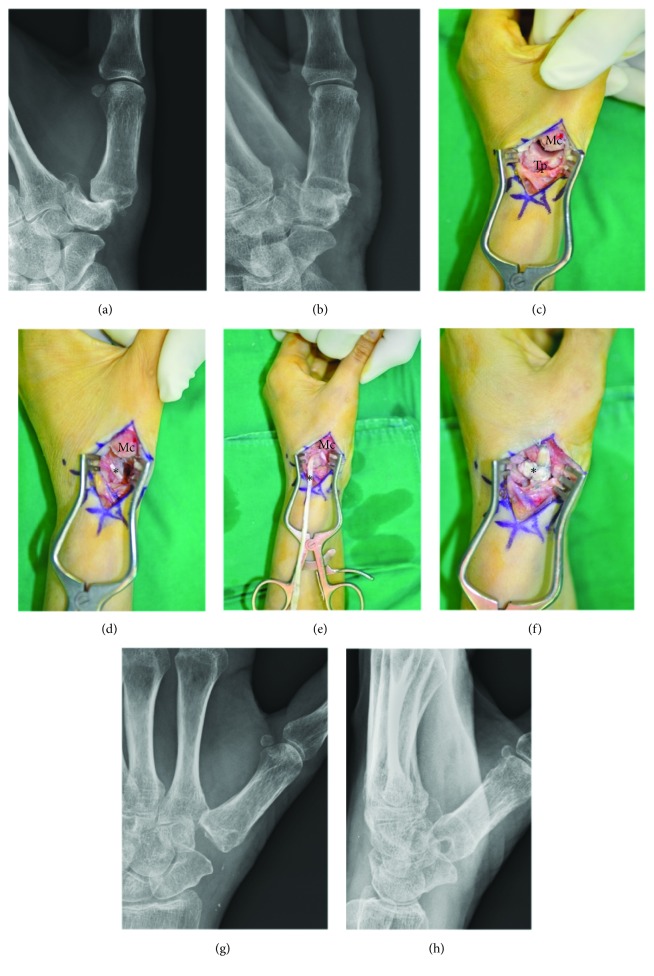
The ligament reconstruction and tendon interposition (LRTI) surgical procedure. (a, b) Posteroanterior and lateral radiographs of a 52-year-old female with Eaton-Littler stage III trapeziometacarpal (TMC) osteoarthritis. (c) After opening the dorsal capsule of the TMC joint, the base of the first metacarpal bone and trapezium were exposed. (d) The base of the insertion of the flexor carpi radialis (FCR) tendon of the second metacarpal bone was noted (*∗*) after excision of the trapezium. Care was taken to avoid FCR tendon injury. (e) The distally based radial half of the FCR tendon was harvested from its musculotendinous junction and retrieved distally to its insertion. (f) The retrieved tendon was delivered through the bone tunnel, and then the FCR remnant was weaved along its length using the anchovy procedure and interposed in the trapezium space and anchored deeply to the joint capsule. (g, h) Posteroanterior and lateral postoperative radiographs. Mc, metacarpal bone; Tp, trapezium.

All surgical procedures were performed by one senior hand surgeon (YRC) using regional or general anesthesia. In both surgical procedures, the limb was exsanguinated with an elastic bandage and a pneumatic tourniquet was inflated. The arm distal to the tourniquet was exposed. In Group L (Figure 1), a longitudinal incision was made on the dorsal side of the TMC joint, and the radial dorsal sensory nerves and the radial artery were identified and protected from injury. The dorsal capsule then was opened longitudinally. The trapezium was excised through an initial sagittal saw cut and broken into small pieces using a small sharp osteotome, and then removed piece by piece. During this procedure, special care was taken to avoid flexor carpi radialis (FCR) tendon injury. The articular surface at the base of the metacarpal of the thumb was then removed, with the sagittal saw directed perpendicular to the long axis of the metacarpal to leave the insertion of the abductor pollicis longus tendon intact. All osteophytes on the volar and ulnar sides of the metacarpal base and radial side of the trapezoid also were resected at this point. An oblique hole then was made in the dorsal metacarpal cortex 1 cm distal to the cut metacarpal base in the plane of the thumbnail, and directed into the trapezial space through the medullary canal using a 3-mm burr. The distally based radial half of the FCR tendon was harvested from its musculotendinous junction and retrieved distally to its insertion on the second metacarpal base with two separate 1.5-cm transverse incisions at the distal wrist crease and mid-forearm. The retrieved tendon was delivered through the bone tunnel with a 26- or 30-gauge monofilament loop wire. After longitudinal traction was applied to the thumb to bring the base level with the second metacarpal, the FCR tendon was pulled tightly to remove any slack and then sutured to the metacarpal periosteum and back onto itself with 4-0 nonabsorbable sutures. Kirschner wire fixation of the first metacarpal to the second metacarpal was used for temporary stabilization. The remaining FCR remnant then was weaved along its length like an anchovy and interposed in the trapezium space and then anchored deeply to the joint capsule. The capsule was closed with nonabsorbable suture material. The skin was closed with 4-0 nylon sutures.

**Figure 2:**
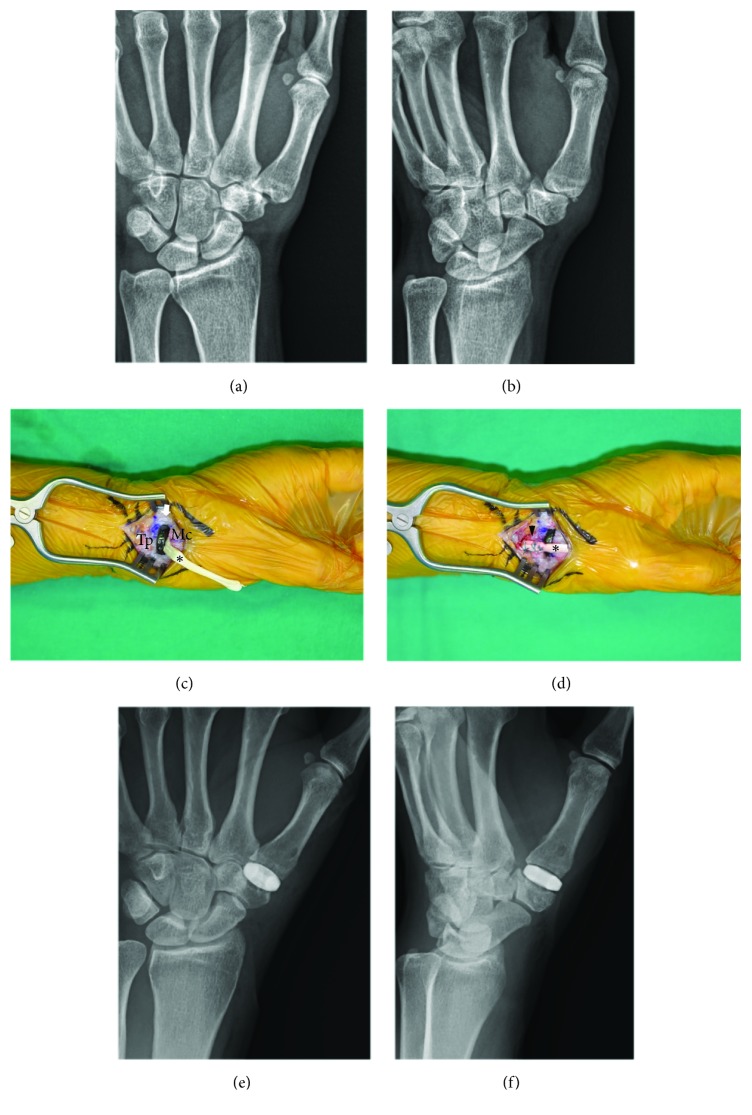
The pyrocarbon interposition arthroplasty surgical procedure. (a, b) Posteroanterior and 45-degree oblique radiographs of a 62-year-old female with Eaton-Littler stage II trapeziometacarpal osteoarthritis. (c) The PyroDisk implant (arrow) was inserted in the trapezium space after resecting both the base of the first metacarpal bone and the distal trapezium using a sagittal saw and 3-mm burr to create concavity. The half flexor carpi radialis (FCR) tendon (*∗*) was passed through the trapezium into the resected joint through the selected PyroDisk implant. (d) The FCR tendon then was continuously passed into the thumb metacarpal bases to exit dorsally through the prepared passage and sutured to the inserted portion of FCR tendon (arrowhead). (e, f) Posteroanterior and 45-degree oblique postoperative radiographs. Mc, metacarpal bone; Tp, trapezium.

In Group P (Figure 2), the same longitudinal incision was made over the TMC joint. A transverse capsulotomy of the TMC joint was performed, and the trapezium was exposed subperiosteally, leaving the capsule for closure. A sagittal saw was used to remove 2 to 3 mm from the thumb metacarpal base perpendicular to the long axis of the metacarpal bone. The sagittal saw also was used to flatten the trapezial saddle using the metacarpal cut for parallel alignment. A 3-mm burr and a reamer were used to create a concavity between the base of the thumb metacarpal and the distal surface of the trapezium. Using the 3-mm burr, a hole in the trapezium was made from the center of the scaphotrapezial joint toward the center of the TMC joint, and the other oblique hole in the thumb metacarpal was made from the dorsal radial aspect of the thumb metacarpal base through the medullary canal as described above. The proper implant diameter was determined by selecting the PyroDisk implant from 6 available sizes that provided the best fit to the diameter of the thumb metacarpal base without overhang. If the best size fell between two available sizes, then the smaller size was chosen. The goal was to achieve a gentle rocking motion of the biconvex disc on the concave surfaces of both the thumb metacarpal base and the trapezium. The distally based radial half of the FCR tendon, which was used as a stabilizer tendon, was retrieved distally—proximal to the trapezium—after it was partially transected at its musculotendinous junction with two separate 1.5-cm transverse incisions at the distal wrist crease and mid-forearm. The distal stump of the FCR tendon then was passed through the trapezium into the resected joint through the selected PyroDisk implant and into the thumb metacarpal bases to exit dorsally through the prepared passage. Gentle traction was applied to the tendon prior to closure to enhance stability. The remaining tendon then was folded back and incorporated into a secure capsular closure. The capsule was closed with nonabsorbable suture material. The skin was closed with 4-0 nylon sutures.

### 2.2. Postoperative Rehabilitation

After surgery, a compressive dressing and postoperative short-arm thumb spica splint were placed, leaving the interphalangeal joint free. All patients were encouraged to initiate immediate digital exercises to reduce swelling. At the first postoperative visit at 2 weeks, a well-molded short-arm thumb spica cast was applied for 3 to 4 additional weeks to hold the wrist in a functional position. After 5-6 weeks, the cast was replaced with a removable short-arm thumb spica orthosis (the K-wire was removed in Group L at this time point), and patients were encouraged to complete intermittent active movement exercises for the next 2 to 3 weeks followed by resumption of gentle daily activities were permitted. After 12 weeks, patients started pinch and grasp strengthening exercises and unrestricted activities were allowed. Patients had regular follow-ups at an outpatient clinic at 6 months, 1 year, and annually thereafter.

### 2.3. Subjective and Objective Outcome Measurements

One observer (HNC) not involved in the treatment performed preoperative and postoperative assessments using a VAS pain score of 0 to 10, the Kapandji score, grip and key pinch strengths, and DASH scores. The Kapandji score was used to assess thumb opposition. This score is determined from the location on their hand that the patient is able to touch with the tip of their thumb from 1 (radial side of the proximal phalanx of the index finger) to 10 (distal palmar crease of the little finger) [14]. Grip strength was measured using a JAMAR hydraulic dynamometer (Asimov Engineering, Los Angeles, CA, USA) and key pinch strength was measured using a pinch gauge (B&L Engineering, Tustin, CA, USA). The DASH questionnaire, a self-reported questionnaire introduced by Hudak et al. [15], was administered to each patient preoperatively and at each follow-up. The questionnaire contains 30 items: 21 questions that assess difficulties with specific tasks, 5 questions that evaluate symptoms, and 4 questions that evaluate social function, work function, sleep, and confidence. DASH scores range from 0 to 100 with higher scores representing greater upper extremity disability.

Each patient also was assessed for any surgery-related or other complications throughout the follow-up period.

### 2.4. Radiographic Measurements

**Figure 3:**
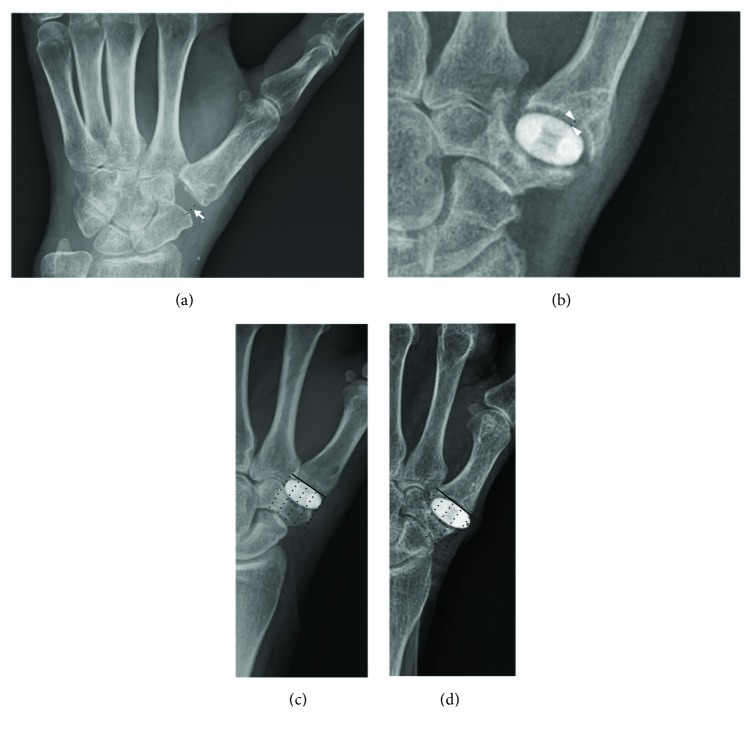
Postoperative radiographic measurements. (a) Proximal migration of the first metacarpal bone (arrow) was measured as the distance between the distal articular surface of the scaphoid and the proximal articular surface of the first metacarpal bone. (b) Radiolucency around the implant (arrowhead) was measured between the edge of the implant and the most distant rim of the surrounding metacarpal bone or trapezium. (c) Implant migration (*∗*) was measured based on divided quarters of the trapeziometacarpal joint and classified as centered, one-fourth displaced, one-half displaced, or greater than one-half displaced.

To calculate the degree of proximal migration of the first metacarpal in both groups, immediate postoperative radiographs were compared with the true lateral radiographs of the thumbs taken with the thenar area on the radiograph cassettes by deducting the distances between the distal articular surface of the scaphoid and the proximal articular surface of the first metacarpal (Figure 3(a)). Coronal and sagittal implant alignment in relation to the long axis of the TM joint was measured as described by Barrera-Ochoa et al. [10]. The base of the TM joint was divided into quarters on the frontal and lateral views. Implant migration (subluxation) (Figures 3(c) and 3(d)); radiolucency around the implant (Figure 3(b)), which provides evidence for fractures or skeletal erosions; and the development of arthritis at neighboring joints also were evaluated. Implant subluxation at the last follow-up was classified as centered, one-fourth displaced, one-half displaced, or greater than one-half displaced [10]. Measurements from these radiographs were assessed twice by the same independent orthopedic surgeon (WTO); an average of these two measurements was used in the analysis.

### 2.5. Statistical Analysis

SPSS Statistics version 23.0 (SPSS, Inc., IBM®, Chicago, IL, USA) was used for all statistical analyses. Group results were compared using either the Pearson's chi-squared test or Fisher's exact test for the categorical variables and the Student's t-test or the Wilcoxon rank-sum test for the continuous variables. For all analyses, the level of significance was set at p<0.05. Power analysis, which was performed with key pinch strength at last follow-up, found that power was 0.98 with the following values for independent* t*-tests: sample sizes: 19 (group I) and 20 (group II); effect size, 1.26; and standard deviation, 1.43. To determine the minimum number of subjects for adequate study power, we estimated with DASH score setting the minimally clinical important difference as 15 points. The minimum sample size that can have sufficient statistical power to detect a treatment effect was 10 (effect size, 15.0; *α*, 0.05; *β*, 0.2; power, 0.8)

## 3. Results

Patient demographics did not differ significantly between the patient groups (Table 1). Patient ages ranged from 50 to 72 years in Group L and from 48 to 73 years in Group P. Two patients in Group L and one patient in Group P underwent bilateral surgery for advanced TMC arthritis. Of these patients, only the dominant extremity was analyzed.

The mean VAS pain score improved significantly from 6.5(SD 1.6) preoperatively to 0.7 (SD 1.0) at the last follow-up evaluation in Group L (p<0.001) and from 5.8 (SD 1.9) to 0.8 (SD 1.0) in Group P (p<0.001). The postoperative VAS pain scores at the last follow-up were similar between the two groups (p=0.757). Grip and key pinch strength also improved significantly from 20.3 (SD 6.0) kg and 6.7 (SD 1.7) kg, respectively, preoperatively to 28.4 (SD 5.1) kg and 8.9 (SD 1.1) kg, respectively, at the last follow-up in Group L (p<0.001 and <0.001) and from 23.2 (SD 10.6) kg and 6.6 (SD 2.6) kg, respectively, to 32.7 (SD 8.1) kg and 10.7 (SD 1.7) kg, respectively, in Group P (p<0.001 and <0.001). The grip strength at the last follow-up was similar between the two groups (p=0.082), but the key pinch strength was significantly higher in Group P compared to Group L (p<0.001). The mean Kapandji score also improved significantly from 7.7 (SD 1.4) preoperatively to 9.2 (SD 0.9) at the last follow-up in Group L (p<0.001) and from 7.7 (SD 0.9) to 9.1 (SD 0.6) in Group P (p<0.001). The difference in the Kapandji score at the last follow-up was similar between the two groups (p=0.429). Mean DASH scores improved significantly from 47.4 (SD 11.9) preoperatively to 7.9 (SD 4.4) at the last follow-up in Group L (p<0.001) and from 55.6 (SD 20.5) to 8.7 (SD 4.4) in Group P (p<0.001). DASH scores at last follow-up were similar between the two groups.

Mean proximal metacarpal migration did not differ significantly between the two groups (1.4 (SD 0.2) mm in Group L and 1.9 (SD 0.2) mm in Group P, p=0.883). The implant displaced one-fourth of the implant diameter in three wrists and tilted significantly in one wrist. Periprosthetic lucency of the implant was more than 1 mm in seven wrists (Table 2).

There was one case of transient neuropraxia of the superficial radial nerve in each group, which resolved within 6 weeks postoperatively. In one case of Group L, the FCR tendon was completely ruptured during complete trapezium excision. The radial half of the extensor carpi radialis longus was harvested to finish the LRTI procedure [16]. No revision surgery was reported in either group.

The average of operative times showed no statistical difference between Group L (90.2 ± 20.0 minutes) and Group P (84.4 ± 15.1 minutes). The average operative room cost was higher in Group P ($11,281 ± 624) than Group L ($10,023 ± 812) due to the cost of the implant ($1,000).

## 4. Discussion

Once radiographs show advanced TMC osteoarthritis, partial or complete trapezium resection with or without interposition or prosthesis implantation are surgical options to relieve pain and improve function while preserving joint movement when nonoperative treatment fails. Controversy exists, however, regarding the surgical method that best improves pain and functional outcomes. This study compared clinical and radiological outcomes and complications after PyroDisk interpositional arthroplasty to trapezium excision with LRTI, and found similar clinical outcomes on measures of pain, function, and grip strength. Key pinch strength at a minimum follow-up of 2 years was the only significantly different clinical outcome between LRTI and PyroDisk interpositional arthroplasty. Long-term follow-up of periprosthetic changes after PyroDisk interpositional arthroplasty is recommended, although the changes observed in this study were not correlated with any clinical outcomes.

Our study has several limitations. First, the small sample size resulted in low statistical power, thereby increasing the chance of type II errors in our statistical findings. Second, there may have been selection bias in patient enrollment for each surgical procedure. During the study period, one experienced hand surgeon alternated between the two surgical procedures when patients met the inclusion criteria during preoperative evaluation. Third, all patients in our study except one were female. This sex distribution is not unusual because basal thumb arthritis is reported to occur predominantly in postmenopausal women and in a female:male ratio of 6:1 [17, 18]. Furthermore, arthrodesis could be the treatment of choice in patients with high demands places, especially in men. Fourth, the follow-up period was insufficient to determine the relationship between the development of periprosthetic changes after PyroDisk arthroplasty and functional outcomes. Longer follow-up periods will be required to evaluate this relationship. Finally, this study was retrospective. A prospective randomized design is required to confirm the efficacy of PyroDisk arthroplasty over LRTI after trapezium excision.

The ideal goal in treating TMC arthritis is to relieve pain while preserving stable joint movement and retaining or improving grip and pinch strength. Various arthroplasty techniques that have been developed can be categorized into two groups based on the material interposed between the resected joint: autologous tendon versus artificial implant [19]. Pellegrini and Burton modified the technique of trapezium excision and FCR anchovy tendon interposition described by Froimson by combining ligament reconstruction of the TMC joint to enhance joint stability [4, 5]. Although simple trapeziectomy has recently regained popularity [4, 5], the LRTI technique is now the most popular surgical technique for TMC arthritis with successful and robust outcomes [19–21]. Issues regarding weakness in pinch strength with LRTI, due to shortening or instability after complete trapezium excision, have been raised, however. Although the increase in pinch strength observed in our study after LRTI (from 8% to 17%) is similar to other studies [5, 6], this increase is less than the increase in pinch strength observed after PyroDisk arthroplasty. Previous studies have raised the issue of subsidence of the thumb metacarpal after LRTI and its role on the limited improvement of pinch strength. Based on serial radiographic measurements from other studies, the metacarpal settles by 11% (to 33%) after LRTI and subsides another 10.5% with powerful lateral pinch [4, 6, 22]. This subsidence is one reason why implant arthroplasty has been attempted in patients with TMC arthritis. Mechanical studies have shown that prosthesis implantation reduces metacarpal subsidence and better replicates normal joint motion [7–9]. Implant arthroplasty has been criticized, however, due to high rates of failure due to breakage, instability, loosening, and stiffness. Previous studies have not reported superiority of implant arthroplasty to other techniques in terms of postoperative pain and function, range of movement, and strength [23–25]. At a minimum follow-up of 5 years, the overall survival of the implant has been reported to range from 85% to 95% [23–25].

The elastic modulus of pyrolitic carbon is similar to cortical bone when compared to other materials, such as cobalt-chrome, titanium, or zirconia [26]. Some pyrolytic carbon implants have been used to restore biomechanics in patients with advanced thumb basal joint arthritis due to pyrolytic carbon's biomechanical compatibility with bone [10–12, 27, 28]. In the past, NuGrip and Pi2 pyrocarbon implants were associated with low satisfaction and high dislocation rates of 29% and 33%, respectively [27, 28]. By making a central hole and incorporating a ligament reconstruction to stabilize the implant, PyroDisk implants achieve stability without early implant dislocation and with better clinical outcomes in terms of pain relief, range of movement, and pinch and grip strength as well as low rates of complications [10, 11]. In our study, pain, grip strength, opposition, and DASH scores were improved similarly after trapezium excision with LRTI and PyroDisk arthroplasty at a minimum follow-up of 2 years. Key pinch strength, however, was significantly higher after PyroDisk arthroplasty compared to LRTI (p<0.001). Interestingly, mean proximal metacarpal migration did not differ significantly between the two groups (1.4 mm after LRTI and 1.9 mm after PyroDisk arthroplasty), which suggests that thumb length alone may not determine key pinch strength after thumb basal joint reconstruction. It is well known that joint reaction force at the TMC joint is biomechanically 12 times greater than that generated at the tip of the thumb via a key pinch [29], indicating that joint reaction during a key pinch is one determining factor in the generation of normal key pinch strength. We believe that PyroDisk arthroplasty generates better joint reaction force and key pinch strength due to the insertion of a solid construct proximal to the metacarpal bone, in contrast to LRTI in which the trapezium space is filled with soft tissue construct. Repetitive movement of the reconstructed thumb during daily activities inevitably causes radiographic changes around the implant after any kind of artificial joint replacement. In our study, the implant displaced one-fourth of the implant diameter in three cases and tilted significantly in one. Periprosthetic lucency of the implant was more than 1 mm in seven cases, but did not correlate with frank implant loosening or failure with a minimum of 2 years follow-up, which is consistent with observations by Barrera-Ochoa et al.[10]. Recent studies of the trapeziometacarpal joint arthroplasty have reported 89% to 95% of survival rate at 5 years from the operation, and revision rate was 0% to 11.5%[30, 31]. The long-term comparative analysis should be required to determine which arthroplasty implant and surgical treatment have superior durability and failure rate.

The revision rate after PyroDisk arthroplasty has been reported to be between 2.8% to 10% for persistent pain or late onset instability [10, 11]. Interestingly, Barrera-Ochoa et al. reported two revisions conducted in patients with symptomatic displaced implants who already had experienced displaced implants postoperatively [10]. In our study, no revisions were necessary in the PyroDisk arthroplasty group at a minimum follow-up of 2 or more years. As previously recommended [10], proper sizing and initial positioning of the implants is important to resolve patient symptoms and increase implant longevity. Surgeons should pay careful attention to center the metacarpal and trapezium bone tunnels and position a properly sized implant. If the proper size falls between two available sizes, we prefer the smaller size to prevent the implant from irritating and attenuating the abductor pollicis longus tendon.

Anatomically, the trapezium transverses the FCR tendon in a deep groove on the palmar surface, putting the FCR tendon at risk during trapezium excision. One safe method is to remove the trapezium piece by piece after breaking it into small pieces using a small sharp osteotome after incomplete sawing. We experienced one complete rupture of the FCR tendon during complete trapezium excision during the LRTI procedure. In that case, the radial half of the distally based extensor carpi radialis longus tendon was harvested and used to replace the role of the FCR tendon. King et al. previously described the LRTI technique using the extensor carpi radialis longus tendon based on the anatomically close relationship between the tendon and the intermetacarpal ligament [16]. Alternately, the abductor pollicis longus tendon, initially described by Thompson, could be used in this situation [32, 33].

## 5. Conclusions

All subjective and objective outcomes were similar following LRTI and pyrolytic interpositional arthroplasty in patients with TMC arthritis, except pinch strength, which was more improved following pyrolytic interpositional arthroplasty. Longer follow-up is required to test adverse effects of high rates of periprosthetic lucency and prosthetic subluxation on clinical outcomes after PyroDisk interpositional arthroplasty.

## Figures and Tables

**Table 1:** Patient demographics.

Variables	Group L	Group P	P value∗
	(n = 19)	(n = 20)	
Age (years)	58.9 (SD 6.4)	63.3 (SD 8.4)	0.091
Male / Female (n)	0 / 19	1 / 19	1.000
Dominant / Non-dominant (n)	5 / 14	9 /11	0.378
Symptom duration (months)	59.4 (SD 10.9)	57.0 (SD 14.0)	0.671
VAS pain score	6.5 (SD 1.6)	5.9 (SD 1.9)	0.130
Grip strength (kg)	20.3 (SD 6.0)	23.2 (SD 10.6)	0.253
Pinch strength (kg)	6.7 (SD 1.7)	6.6 (SD 2.6)	0.819
Kapanji score	7.7 (SD 1.4)	7.7 (SD 0.9)	0.953
DASH score	47.4 (SD 11.9)	55.6 (SD 20.5)	0.220
Follow-up period (months)	40.6 (SD 21.2)	35.2 (SD 15.2)	0.475

Continuous variables are reported as the mean (standard deviation). Group L = trapezium excision with ligament reconstruction and tendon interposition (LRTI); Group P = PyroDisk interpositional arthroplasty. ∗P values were computed from Student's t-test or the Wilcoxon rank-sum test for continuous variables and Pearson's chi-squared test or Fisher's exact test for categorical variables. VAS, visual analog scale; DASH, disabilities of arm, shoulder, and hand.

**Table 2:** Clinical and radiologic outcomes at last follow-up.

Variables	Group L	Group P	P value∗
	(n = 19)	(n = 20)	
VAS pain score	0.7 (SD 1.0)	0.8 (SD 1.0)	0.757
Grip strength (kg)	28.4 (SD 5.1)	32.7 (SD 8.1)	0.082
Pinch strength (kg)	8.9 (SD 1.1)	10.7 (SD 1.7)**†**	< 0.001
Kapanji score	9.2 (SD 0.9)	9.1 (SD 0.6)	0.429
DASH score	7.9 (SD 4.4)	8.7 (SD 4.4)	0.283
Proximal metacarpal migration (mm)	1.4 (SD 0.2)	1.9 (SD 0.2)	0.883
Implant subluxation (n)			
Centered	-	17	
<1/4 displaced	-	3	
<1/2 displaced	-	0	
>1/2 displaced	-	0	
Peri-prosthetic lucency			
<1 mm	-	19	
>1 mm	-	1	

Continuous variables are reported as the mean (standard deviation). Group L = trapezium excision with ligament reconstruction and tendon interposition (LRTI); Group P = PyroDisk interpositional arthroplasty. ∗P values were computed from Student's t-test or the Wilcoxon rank-sum test for continuous variables and Pearson's chi-squared test or Fisher's exact test for categorical variables. †P<0.05 VAS, visual analog scale; DASH, disabilities of arm, shoulder, and hand.

## Data Availability

The patients' data used to support the findings of this study have been deposited in the WTO's repository (owont@yuhs.ac).
